# Efficacy and safety of the recombinant human growth hormone in short children born small for gestational age: A randomized, multicentre, comparative phase III trial

**DOI:** 10.1097/MD.0000000000026711

**Published:** 2021-07-30

**Authors:** Su Jin Kim, Min-Sun Kim, Sung Yoon Cho, Byung-Kyu Suh, Cheol Woo Ko, Kee-Hyoung Lee, Han-Wook Yoo, Choong Ho Shin, Jin Soon Hwang, Ho-Seong Kim, Woo Yeong Chung, Chan Jong Kim, Heon-Seok Han, Dong-Kyu Jin

**Affiliations:** a Department of Pediatrics, Inha University College of Medicine, Inha University Hospital, Incheon, Korea; b Department of Pediatrics, Samsung Medical Center, Sungkyunkwan University School of Medicine, Seoul, Korea; c Department of Pediatrics, Seoul St. Mary's Hospital, The Catholic University of Korea, Seoul, Korea; d Department of Pediatrics, Kyungpook National University Hospital, Daegu, Republic of Korea; e Department of Pediatrics, Korea University Anam Hospital, Seoul, Korea; f Department of Pediatrics, Medical Genetics Clinic and Laboratory, Asan Medical Center Children's Hospital, University of Ulsan College of Medicine, Seoul, Korea; g Department of Pediatrics, Seoul National University Children's Hospital, Seoul, Korea; h Department of Pediatrics, Ajou University Hospital, Suwon, Korea; i Department of Pediatrics, Severance Hospital, Yonsei University College of Medicine, Seoul, Korea; j Department of Pediatrics, Inje University Busan Paik Hospital, Busan, Korea; k Department of Pediatrics, Chonnam National University Hospital, Gwangju, Korea; l Department of Pediatrics, Chungbuk National University Hospital, Cheongju, Korea.

**Keywords:** recombinant human growth hormone, short stature, small for gestational age

## Abstract

**Objective:**

Growth hormone (GH) treatment is known to be effective in increasing stature in children with a short stature born small for gestational age (SGA). This multicentre, randomized, open-label, comparative, phase III study aimed to evaluate the efficacy and safety of Growtropin-II (recombinant human GH) and to demonstrate that the growth-promoting effect of Growtropin-II is not inferior to that of Genotropin in children with SGA (NCT ID: NCT02770157).

**Methods:**

Seventy five children who met the inclusion criteria were randomized into 3 groups in a ratio of 2:2:1 (the study group [Growtropin-II, n = 30], control group [Genotropin, n = 30], and 26-week non-treatment group [n = 15]). The study and control groups received subcutaneous injections of Growtropin-II and Genotropin, respectively for 52 weeks, whereas the non-treatment group underwent a non-treatment observation period during weeks 0 to 26 and a dosing period during weeks 27 to 52 and additional dosing till week 78 only in re-consenting children.

**Results:**

No significant differences in demographic and baseline characteristics between the groups were observed. The mean ± standard deviation change difference in annualized height velocity (aHV) (study group - control group) was 0.65 ±2.12 cm/year (95% confidence interval [CI], −0.53 to 1.83), whereas the lower limit for the 2-sided 95% CI was −0.53 cm/year. Regarding safety, treatment-emergent adverse events (TEAEs) occurred in 53.33% children in the study group and 43.33% children in the control group; the difference in the incidence of TEAEs between the 2 treatment groups was not statistically significant (*P* *=* .4383). A total of 17 serious adverse events (SAEs) occurred in 13.33% children in the treatment groups, and no significant difference in incidence between groups (*P* *=* .7065) was seen. Two cases of adverse drug reaction (ADR) occurred in 2 children (3.33%): 1 ADR (injection site swelling or pain) occurred in 1 child (3.33%) each in the study and control groups.

**Conclusions:**

This study demonstrates that the change in aHV from the baseline till 52 weeks with Growtropin-II treatment is non-inferior to that with Genotropin treatment in children with short stature born SGA. Growtropin-II is well-tolerated, and its safety profile is comparable with that of Genotropin over a 1-year course of treatment.

## 1 Introduction

Children born small for gestational age (SGA) are those with birth weights less than the third percentile or 2 standard deviation scores (SDS) less than the mean compared to all babies of the same gestational age and sex.^[1]^ Children born SGA are a heterogeneous group at risk for short adult stature and metabolic and endocrine consequences. About 85% to 90% of infants born SGA reach an age-appropriate height and/or weight through catch-up growth within 2 years.^[2,3]^ However, 10% to 15% of such infants, do not experience rapid catch-up growth and remain short as adults.^[4]^ The efficacy and safety of growth hormone (GH) treatment in children born SGA with short stature has been reported in several studies.^[5–7]^ Short stature is only one of the challenges such individuals face. According to epidemiological studies, children born SGA tend to have low lean body mass and increased central adiposity,^[8]^ and are at risk of cardiovascular conditions in later life.^[9]^ In addition, low birth weight is associated with a higher prevalence of metabolic risk factors including insulin resistance and type 2 diabetes in adulthood.^[10,11]^ These conditions should be considered when contemplating GH treatment in children born SGA with short stature. GH treatment for children with short stature born SGA who are aged >4 years and without evidence of catch-up growth has been covered by National Health Insurance in Korea since 2014.^[12]^ A few reports have examined the efficacy and safety of GH treatment in Korean children born SGA.^[13,14]^ The present study is a 1-year, multicentre, randomized controlled, open-label, comparative, phase III study of Korean children with short stature born SGA. This study aims to

1. evaluate the efficacy and safety of recombinant human GH (Growtropin-II) and
2. demonstrate that the growth-promoting effect of Growtropin-II is not inferior to that of Genotropin.

## 2 Methods

### 2.1 Study design and patients

This is a multicentre, randomized, open-label, parallel-group, comparative, phase III study. The inclusion criteria were as follows: pre-pubertal children; evaluated according to the Tanner classification: testicular volume of 4 cm^3^ or less in boys and Tanner stage I breast development in girls, with a chronological age (CA) of ≥4 years; height below the third percentile among the Korean population of the same sex and age; availability of official stature records of at least up to 6 months before the start of the trial; gestational age at birth of 23 to 43 weeks and weight at birth below the third percentile among the same gestational age group; naïve to GH therapy; and normal thyroid function (or normalized after hormone therapy). The exclusion criteria were as follows: a medical history of a condition other than SGA that may cause growth delay (e.g., Turner syndrome, Prader-Willi syndrome, Down syndrome, Noonan syndrome, Russell-Silver syndrome, and other chromosomal abnormalities); clinically significant congenital or acute or chronic disease; hypersensitivity to GH preparations; and present use of drugs that interfere with secretion and action of GH, such as oestrogens, androgens, anabolic steroids, corticosteroids or methylphenidate. The study protocol and consent form were reviewed by the Institutional Review Board of each institution. Before the screening test, informed consent was obtained from the participants themselves and their legally authorized representatives (e.g., parents). This study was registered at clinicaltrials.gov (identifier: NCT02770157).

### 2.2 Methods

**Figure 1:**
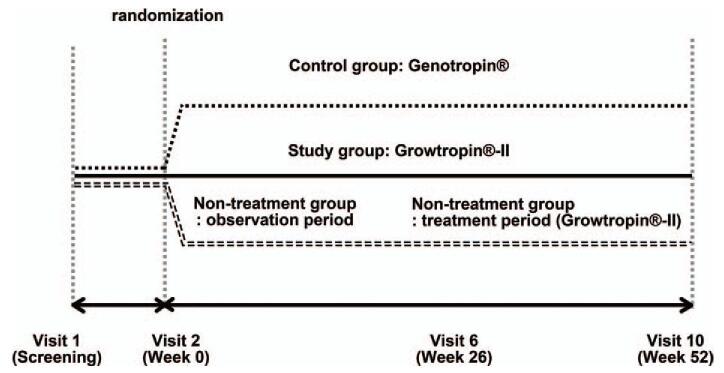
Schematic representation of the study design.

Patients who met the inclusion criteria and were eligible for the study based on the screening test (weeks −4 to 0) were centrally randomized 2:2:1 to the study group, control group, or 26-week non-treatment group using a block randomization method. The study and control groups were administered recombinant human GH at a dose of 0.48 mg (1.44 IU)/kg/week, divided into 6 to 7 doses per week; treatment consisted of Growtropin-II (Dong-A ST Co. Ltd., Seoul, Korea) and Genotropin (Pfizer Korea), respectively, for a total study period of 52 weeks. The non-treatment group underwent a non-treatment observation period during weeks 0 to 26 and a dosing period (Growtropin-II, at the same dosage) during weeks 27 to 52 (Fig. 1). Each participant visited the clinic during weeks 13, 26, 39, and 52 to undergo efficacy and safety tests, including measurements of height and weight; assessments of insulin-like growth factor 1 (IGF-1), insulin-like growth factor binding protein 3 (IGFBP-3) and haemoglobin A1c levels; thyroid function tests; and other laboratory tests. IGF-1 levels were measured using immunoradiometric assay with a Dream GAMMA-10 Counter (Shinjin Medics) and Somatomedin-C (ImmunoTek) in our central laboratory. IGFBP-3 levels were measured using chemiluminescence immunoassay with IMMULITE 2000 XPi (Siemens) and directly with IMMULITE 2000 system (Siemens) in our central laboratory. Patients also filled questionnaires regarding medication compliance and adverse events while visiting the institution or via phone interview at the halfway time points of each visit (weeks 6, 19, 32, and 45). In the non-treatment group, individuals who re-consented received Growtropin-II for an additional 26 weeks after the completion of the clinical trial. During those additional 26 weeks, they underwent follow-up visits for safety assessment purposes. Pubertal stage was evaluated by pediatric endocrinologists on each visit. BA and anti-GH antibody levels were determined at the baseline and at weeks 26 and 52. GH antibody levels were measured using a Human Anti-GH antibody immunoassay (MyBioSource) and an ELx808 Absorbance Microplate Reader (BioTeck) at our central laboratory. Bone age (BA) was assessed by a single independent pediatric endocrinologist using the Greulich-Pyle method.

### 2.3 Outcome variables

The difference in annualized height velocity (aHV, cm/year) at week 52 between the study and control groups was considered the primary efficacy endpoint. The difference in aHV (ΔaHV) after week 26 between the control group and the non-treatment group, and the difference in height standard deviation score (Ht-SDS), skeletal maturity (ΔBA/ΔCA), IGF-1 and IGFBP-3 levels at week 52 between the study and control groups were the secondary efficacy points. Ht-SDS was calculated using the growth standard for the Korean population, and IGF-1 SDS was calculated based on normative data on Korean adolescents.^[15]^ Treatment-emergent adverse events (TEAEs) were standardized according to the system organ class and preferred term per the MedDRA software (ver. 22.0; International Federation of Pharmaceutical Manufacturers Associations, https://www.meddra.org).

### 2.4 Statistical analysis

Statistical analyzes were performed using SAS statistical software (ver. 9.4, SAS Institute, Cary, NC, USA). Descriptive statistics are presented as mean ± SD for continuous variables and as frequency and percentage for categorical variables. For the primary efficacy endpoint, if the lower limit of the two-sided 95% confidence interval (that is, one-sided 97.5% confidence interval) for the inter-group difference in the growth rate changes from baseline (before dosing) at week 52 after dosing between the study group (DA-3002) and the control group (Genotropin Inj.) was greater than −1.9, the test drug (DA-3002) was deemed not-inferior to the reference drug (Genotropin Inj.). Differences in efficacy between the study and control groups were analyzed using a 2-sample t test or the Wilcoxon rank sum test for continuous data and chi-square test or Fisher exact test for categorical data. TEAEs were also summarized by severity and relationship to the study drug. Laboratory test results were classified as normal or abnormal according to the normal range, and the McNemar test was used to compare changes before and after administration of the investigational products. A *P* value <.05 was considered statistically significant.

## 3 Results

### 3.1 Patient characteristics at baseline

**Figure 2:**
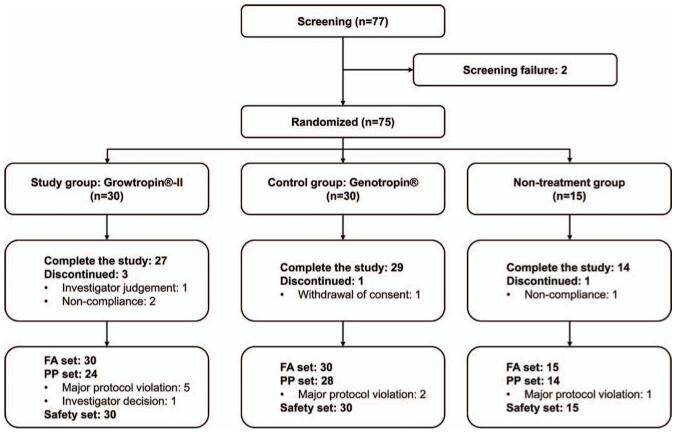
Flow of the distribution of subjects.

In total, 77 patients were recruited from 11 centers in February 2016, of whom 75 were enrolled in this study. The study ended in August 2019. The 75 patients were randomized 2:2:1 to the study group, control group and 26-week non-treatment group. Five participants (6.67%) opted out of the study, leaving 70 participants who completed the study (Fig. 2). The 75 patients included 37 boys (49.3%) and 38 girls (50.7%). The mean CA of the participants was 5.75 ± 1.68 years. Their mean height and body weight were 101.26 ± 9.77 cm and 15.27 ± 3.64 kg, respectively, and their mean BMI was 14.70 ± 1.28 kg/m^2^. Their mean gestational age and weight at birth were 37.79 ± 3.12 weeks and 2198.53 ± 593.44 g, respectively. The baseline characteristics or other demographic and auxologic data did not differ significantly among the groups (Table 1).

**Table 1:** Baseline and demographic characteristics of the study subjects.

	Study group (Growtropin-II) (n = 30)	Control group (Genotropin) (n = 30)	Non-treatment group (n = 15)	Total (n = 75)
Male/female	12/18	15/15	10/5	37/38
Chronological age (yr)	6.00 ± 0.99	5.47 ± 4.39	5.82 ± 8.56	5.75 ± 7.68
Bone age (yr)	4.56 ± 1.64	4.22 ± 1.12	5.13 ± 1.78	4.55 ± 1.51
Height (cm)	102.28 ± 21.43	99.71 ± .11	102.32 ± 2.45	101.26 ± 1.77
Height-SDS	−2.61 ± 0.79	−2.59 ± 0.63	−2.46 ± 0.64	−2.57 ± 0.69
Weight (kg)	15.75 ± .46	14.60 ± .77	15.65 ± .36	15.27 ± .64
BMI (kg/m^2^)	14.77 ± 1.33	14.58 ± 1.24	14.78 ± 1.35	14.70 ± 1.28
BMI-SDS	−1.00 ± 1.23	−1.05 ± 1.15	−0.97 ± 1.36	−1.01 ± 1.21
GA (wk)	38.07 ± .02	37.63 ± .63	37.53 ± .24	37.79 ± .12
Weight at birth (g)	2271.33 ± 760.20	2137.33 ± 371.71	2175.33 ± 717.23	2198.53 ± 993.44
Pretreatment height velocity (cm/yr)	5.50 ± 1.40	5.70 ± 2.04	5.43 ± 1.03	5.56 ± 1.61

### 3.2 Efficacy results

**Figure 3:**
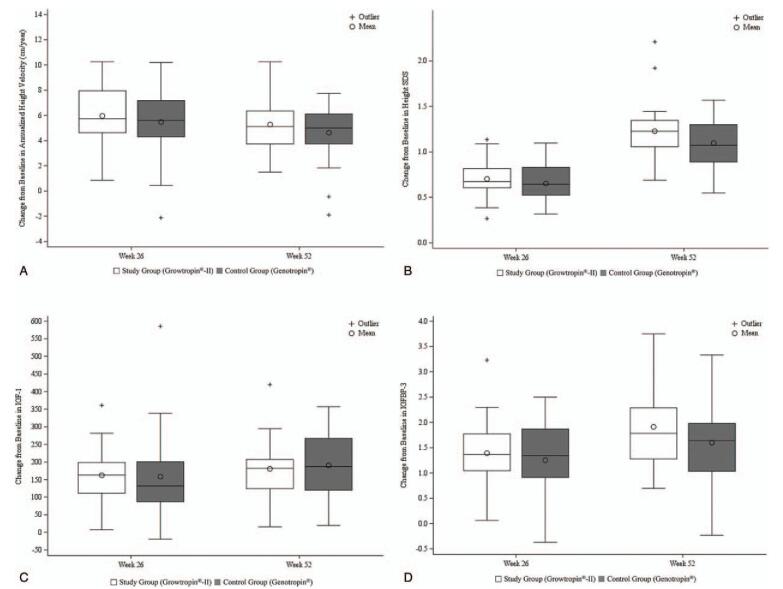
Differences in change from baseline in annualized height velocity, height SDS, IGF-1 level, and IGFBP-3 level at weeks 26 and 52 in the study group (Growtropin-II) and control group (Genotropin). Change from baseline in (A) annualized height velocity, (B) height SDS, (C) IGF-1 level, and (D) IGFBP-3 level. IGF-1 = insulin-like growth factor-1, IGFBP-3 = insulin-like growth factor binding protein-3, SDS = standard deviation score.

The full analysis (FA) set was defined as the set of all participants who received at least 1 dose of recombinant human GH and underwent at least 1 primary efficacy endpoint measurement after randomization (study: control: non-treatment, 30:30:15). Of the FA set, 66 patients who completed the clinical trial without being lost to follow-up and who committed no major protocol violations were included in the per-protocol (PP) set (study: control: non-treatment, 24:28:14). The PP set was used as the primary set for efficacy evaluation (Fig. 2). The ΔaHV values at week 52 for the Growtropin-II (study) and Genotropin (control) group were 5.28 ± 2.03 cm/year and 4.63 ± 2.19 cm/year, respectively (Fig. 3A). The inter-group difference was 0.65 ± 2.12 cm/year (95% confidence interval [CI], −0.53 to 1.83), and the lower limit of the 2-sided 95% CI was −0.53 cm/year, which was greater than the non-inferiority margin of −1.9 (Table 2). These results indicate that the growth-promoting effect of a 52-week administration of Growtropin-II in children with short stature born SGA is non-inferior to that of Genotropin. The change in aHV at week 26 was 5.48 ± 2.61 cm/year in the control group and 0.75 ± 1.30 cm/year in the non-treatment group, which is a significant difference (*P* *<* .0001). Both the study and control groups had significant increases in Ht-SDS from the baseline at weeks 26 and 52 (all *P* *<* .0001). The changes in Ht-SDS at each visit were not significantly different between the study group and the control group (weeks 26 and 52: *P* *=* .3583 and *P* *=* .1894, respectively; Table 2, Fig. 3B). The differences in skeletal maturity (ΔBA/ΔCA), IGF-1 (ΔIGF-1), and IGFBP-3 (ΔIGFBP-3) from the baseline to weeks 26 and 52 were not significantly different between the study group and the control group (Table 2, Fig. 3C and D). The results of the analysis on the efficacy endpoints for the FA set were similar to those for the PP set (data not shown).

**Table 2:** Efficacy results in both treatment groups (per-protocol set).

	Study group (Growtropin-II) (n = 24)	Control group (Genotropin) (n = 28)	*P* value
ΔaHV at Wk 52 (cm/year)	5.28 ± 2.03	4.63 ± 6.19	.65 ± 2.12^∗^ [−0.53,1.83]^†^
Ht-SDS			
Baseline	−2.70 ± 0.83	-2.62 ± 0.64	.8472
Wk 26	0.70 ± 0.20	0.65 ± 0.19	.3583
Wk 52	1.23 ± 0.33	1.10 ± 0.28	.1894
Skeletal maturity			
Baseline (BA/CA)	0.76 ± 0.13	0.77 ± 0.13	.8007
Wk 26 (ΔBA/ΔCA)	0.60 ± 0.71	1.00 ± 0.80	.0840
Week 52 (ΔBA/ΔCA)	0.97 ± 0.50	1.11 ± 0.49	.3056
IGF-1 (ng/mL)			
Baseline	108.72 ± 51.82	122.75 ± 52.68	.2364
ΔIGF-1 at Wk 26	162.59 ± 73.76	158.50 ± 116.60	.4246
ΔIGF-1 at Wk 52	180.95 ± 85.84	190.64 ± 87.39	.6896
IGF-1 SDS			
Baseline	-0.81 ± 0.83	-0.53 ± 0.67	.1824
Wk 26	1.61 ± 1.43	1.85 ± 2.03	.5508
Wk 52	1.47 ± 1.21	2.14 ± 1.98	.3399
IGFBP-3 (μg/mL)			
Baseline	3.21 ± 0.55	3.36 ± 0.66	.4045
ΔIGFBP-3 at Wk 26	1.39 ± 0.70	1.26 ± 0.78	.5046
ΔIGFBP-3 at Wk 52	1.91 ± 0.76	1.60 ± 0.84	.1681

### 3.3 Safety results

All 75 subjects in the FA set were included in the safety set (Fig. 2). In the safety set (in all 60 subjects in the study and control groups), a total of 142 adverse events (TEAEs) occurred in 29 participants (48.33%). Seventy six TEAEs occurred in 16 participants (53.33%) in the study group, and 66 TEAEs occurred in 13 participants (43.33%) in the control group. The most common TEAE was infections and infestations, which was reported in 12 participants (40.00%, 52 cases) in the study group and 10 participants (33.33%, 45 cases) in the control group. Two cases of adverse drug reaction (ADR) occurred in 2 participants (3.33%), 1 in each group (3.33%). The incidence of TEAEs (*P* *=* .4383) or ADRs (*P* = 1.0000) did not differ significantly between the study group and the control group (Table 3). The ADRs were categorized as injection site swelling in 1 subject (3.33%, 1 case) in the study group and injection site pain in 1 patient (3.33%, 1 case) in the control group based on the preferred term. The ADRs that occurred were predictable and one-off local ADRs and they were of mild severity. In the safety set, a total of 17 serious adverse events (SAEs) occurred in 8 of the 60 patients (13.33%) in the study and control groups. By treatment group, 6 cases occurred in 3 patients (10.00%) in the study group and 11 cases occurred in 5 patients (16.67%) in the control group; the incidence of SAEs between the treatment groups did not differ significantly (*P* *=* .7065; Table 4). All reported SAEs were unrelated to the investigational product, and the patients recovered without squeal. During the clinical trial period, there were no serious ADRs, no TEAEs leading to permanent discontinuation, and no TEAEs or ADRs resulting in death in either the study group or the control group. Regarding immunogenicity, the results of the GH antibody test were negative at the baseline and at weeks 26 and 52 in both the study and control groups. No unusual findings were observed on the thyroid function test, vital sign tests, or physical examination.

**Table 3:** TEAEs by SOC in the safety set.

	Study group (Growtropin-II) (n = 30)	Control group (Genotropin) (n = 30)	Total (n = 60)	*P* value
	Number of subjects (%) [number of cases]			
TEAEs	16 (53.33%) [76]	13 (43.33%) [66]	29 (48.33%) [142]	.4383
Infections and infestations	12 (40.00%) [52]	10 (33.33%) [45]	22 (36.67%) [97]	
Respiratory, thoracic and mediatinal disorders	7 (23.33%) [9]	3 (10.00%) [6]	10 (16.67%) [15]	
General disorders and administration site conditions	3 (10.00%) [3]	4 (13.33%) [5]	7 (11.67%) [8]	
Gastrointestinal disorders	2 (6.67%) [2]	3 (10.00%) [4]	5 (8.33%) [6]	
Eye disorders	4 (13.33%) [4]	1 (3.33%) [1]	5 (8.33%) [5]	
Skin and subcutaneous disorders	2 (3.37%) [2]	1 (3.33%) [1]	3 (5.00%) [3]	
Congenital, familial and genetic disorders	1 (3.33%) [1]	1 (3.33%) [1]	2 (3.33%) [2]	
Injury, poisoning and procedural complications	1 (3.33%) [1]	1 (3.33%) [1]	2 (3.33%) [2]	
Investigations	0	1 (3.33%) [1]	1 (1.67%) [1]	
Musculoskeletal and connective tissue disorders	1 (3.33%) [1]	0	1 (1.67%) [1]	
Psychiatric dis orders	1 (3.33%) [1]	0	1 (1.67%) [1]	
Renal and urinary disorders	0	1 (3.33%) [1]	1 (1.67%) [1]	

**Table 4:** SAEs by SOC and PT in the safety set.

	Study group (Growtropin-II) (n = 30)	Control group (Genotropin) (n = 30)	Total (n = 60)	*P* value
	Number of subjects (%) [number of events]			
SAEs	3 (10.00%) [6]	5 (16.67%) [11]	8 (13.33%) [17]	.7065
Infections and infestations	0 (00.00%) [0]	3 (10.00%) [7]	3 (5.00%) [7]	
Bronchitis	0	1 (3.33%) [1]	1 (1.67%) [1]	
Chronic tonsillitis	0	1 (3.33%) [1]	1 (1.67%) [1]	
Herpangina	0	1 (3.33%) [1]	1 (1.67%) [1]	
Influenza	0	1 (3.33%) [1]	1 (1.67%) [1]	
Otitis media	0	1 (3.33%) [1]	1 (1.67%) [1]	
Rhinitis	0	1 (3.33%) [1]	1 (1.67%) [1]	
Tonsillitis	0	1 (3.33%) [1]	1 (1.67%) [1]	
Respiratory, thoracic and mediastinal disorders	2 (6.67%) [4]	1 (3.33%) [2]	3 (5.00%) [6]	
Adenoidal hypertrophy	2 (6.67%) [2]	1 (3.33%) [1]	3 (5.00%) [3]	
Tonsillar hypertrophy	2 (6.67%) [2]	1 (3.33%) [2]	3 (5.00%) [3]	
Congenital, familial and genetic disorders	1 (3.33%) [1]	1 (3.33%) [1]	2 (3.33%) [2]	
Atrial septal defect	1 (3.33%) [1]	0	1 (1.67%) [1]	
Hydrocele	0	1 (3.33%) [1]	1 (1.67%) [1]	
Gastrointestinal disorders	0	1 (3.33%) [1]	1 (1.67%) [1]	
Inguinal hernia	0	1 (3.33%) [1]	1 (1.67%) [1]	
General disorders and administration site conditions	1 (3.33%) [1]	0	1 (1.67%) [1]	
Pyrexia	1 (3.33%) [1]	0	1 (1.67%) [1]	

## 4 Discussion

The results of this study show that Growtropin-II is well-tolerated and effective in SGA Korean children with short stature in comparison to Genotropin. Most TEAEs were mild to moderate and resolved completely, and no serious ADRs were observed during the study period. GH treatment for short stature in children born SGA was approved by the food and drug administration in the USA in 2001, and by the European Medicine Agency in Europe in 2003. The food and drug administration has approved Genotropin administration at a dose of up to 0.48 mg/kg/week for children with short stature born SGA. Previous studies demonstrated that long-term GH treatment at a dose of 0.48 mg/kg/week is safe and effective for normalization of adult height.^[6,7,16,17]^ According to the previous studies, we set the GH dose at 0.48 mg/kg/week in both groups.

In this study, the number of dropouts and protocol violations among the 75 randomized participants was relatively low, and the PP analysis group used in the primary efficacy evaluation analysis was representative of the entire study group. Furthermore, compliance with the test drug was high. Thus, the results are reliable. In the Growtropin-II group, the change in aHV after 52 weeks was 5.28 ± 2.03 cm/year and Ht-SDS had significantly increased to 1.23 ± 0.33 cm at week 52. In the control group, the change in aHV after 52 weeks was 4.63 ± 2.19 cm/year and Ht-SDS had significantly increased by 1.10 ± 0.28 cm at week 52. The 95% CI lies within the non-inferior limit of −1.9 cm/year, thus demonstrating the non-inferiority of Growtropin-II to Genotropin regarding the primary efficacy endpoint. In addition, the change in aHV at week 26 after GH (Genotropin) administration was significantly higher than the change in aHV in the non-treatment group (5.48 ± 2.61 cm/year in the control group vs 0.75 ± 1.30 cm/year in the non-treatment group). Ht-SDS had significantly increased at week 52 in both treatment arms, with no significant difference between the 2 groups. The efficacy results of this study are similar to those of previous studies.^[6,7,16–18]^ A 6-month, single-arm study of Korean children born SGA treated with Eutropin (LG Chem. Seoul, Korea) was published in 2011,^[13]^ which reported that the mean change in growth velocity was 5.30 ± 1.84 cm/year at 6 months after administration of Eutropin. In comparison, ours was a randomized, comparative trial with a longer treatment period for efficacy and safety analysis. Several clinical trials have reported on the effects of GH treatment in children with short stature and born SGA. According to meta-analytical reviews of those trials, the overall mean height gain of GH-treated children born SGA was 1.5 SDS (95% CI, 1.04–1.90) from the start to the end of therapy.^[19]^ Furthermore, there is a dose-response relationship between GH and growth in the treatment of children of short stature born SGA.^[20]^

Skeletal maturation at week 52, represented as the change in BA from the baseline over the change in CA from the baseline, was similar across the 2 groups (0.97 ± 0.50 in the study group vs 1.11 ± 0.49 in the control group) during the study period. Previous studies reported skeletal maturation of 0.7 to 1.5 after 1 year of GH treatment in SGA children and indicated that an increase in BA after GH treatment represents a normal progression.^[21,22]^

In SGA children, plasma IGF-1 levels are relatively low at birth, and disturbances in the GH/IGF axis may be associated with insufficient catch-up growth.^[1,23]^ In this study, IGF-1 SDS was relatively low in both groups at the baseline (Table 2). The levels of IGF-I and IGFBP-3 increased in both groups at week 52 after GH treatment. The observed increases in IGF-1 and IGFBP-3 levels were not significantly different between groups and were within the normal range per age-appropriate reference standards.

With regard to safety outcomes, no statistical or clinical differences between the 2 groups in this study were noted, and the TEAEs that occurred during the trial were generally in line with expectations of GH treatment. The most frequent TEAE in this study was infection, and no new safety concerns occurred consequently. Given the increased incidence of glucose metabolism–related abnormalities in children with short stature born SGA, close monitoring of insulin resistance and glucose levels is recommended throughout the treatment period. Previous studies on the influence of GH treatment on glucose metabolism in short SGA children suggest that GH treatment has no major effect on glucose metabolism.^[24,25]^ In this study, mean blood glucose and haemoglobin A1c levels did not change during treatment, and no case of diabetes was seen in either group. These results are reassuring, as they suggest that GH treatment for SGA children with short stature is safe and well-tolerated.

The main limitation of our study is that it was designed as a non-inferiority trial; hence, only a small number of patients were included, and the treatment period was relatively short.

In conclusion, this study demonstrates that the growth-promoting effect of Growtropin-II is comparable to that of Genotropin in children with short stature born SGA when both treatments are given for 1 year. The safety profiles did not differ significantly between the 2 groups. Growtropin-II can be a potential alternative for the treatment of patients with growth disorders including children born SGA. Further studies on long-term efficacy and safety including a larger sample are needed.

## Author contributions

**Conceptualization:** Su Jin Kim, Min-Sun Kim, Sung Yoon Cho, Cheol Woo Ko, Han-Wook Yoo, Choong Ho Shin, Woo Yeong Chung, Heon-Seok Han, Dong-Kyu Jin.

**Data curation:** Byung-Kyu Suh.

**Formal analysis:** Cheol Woo Ko.

**Investigation:** Byung-Kyu Suh.

**Methodology:** Byung-Kyu Suh, Cheol Woo Ko.

**Resources:** Sung Yoon Cho, Chan Jong Kim, Heon-Seok Han.

**Software:** Kee-Hyoung Lee.

**Supervision:** Sung Yoon Cho, Han-Wook Yoo, Choong Ho Shin, Jin Soon Hwang, Woo Yeong Chung, Dong-Kyu Jin.

**Validation:** Kee-Hyoung Lee.

**Visualization:** Kee-Hyoung Lee, Ho-Seong Kim.

**Writing – original draft:** Su Jin Kim, Min-Sun Kim.

**Writing – review & editing:** Min-Sun Kim.
